# An Eye-Tracking Study on the Impact of Green Consumption Values on the Purchase Intention of Bamboo Products Under the Background of “Replacing Plastic with Bamboo”

**DOI:** 10.3390/bs15091162

**Published:** 2025-08-26

**Authors:** Rui Shi, Tongjia Qiao, Chang Liu, Ziyu Chen

**Affiliations:** 1 School of Economics and Management, Yanshan University, Qinhuangdao 066004, China; shirui@ysu.edu.cn (R.S.); q2466955496@163.com (T.Q.); chenziyu0917@163.com (Z.C.); 2 School of Economics and Management, Anhui Polytechnic University, Wuhu 241000, China

**Keywords:** green consumption, purchase intention, product material, green consumption values, eye-tracking experiment, replacing plastic with bamboo

## Abstract

Despite extensive research on green consumption, consumer purchase intentions for bamboo products under China’s “replacing plastic with bamboo” policy remain underexplored, given growing plastic pollution concerns. Research remains focused on established green products (e.g., green agriculture, energy-efficient appliances, new energy vehicles), overlooking consumer behavior and cognition toward emerging bamboo alternatives. This study employs eye-tracking technology to examine purchase intentions and visual attention allocation mechanisms for bamboo versus plastic products, analyzing the role of green consumption values (GCVs). Using a 2 (material: bamboo/plastic) × 2 (GCVs: high/low) mixed design, we recorded fixation duration, fixation count, and heatmaps from 70 participants. Behavioral results revealed significantly higher purchase intention for bamboo products, particularly among high-GCV consumers. Eye-tracking data showed greater visual attention (fixation duration/count) to bamboo products, with high-GCV participants exhibiting significantly stronger attentional bias toward bamboo. Findings demonstrate that bamboo’s eco-friendly attributes enhance both purchase intention and visual attention allocation, validating material salience in green decision-making. High GCVs strengthen automatic attentional bias toward sustainable materials, reinforcing purchase inclinations. This research provides empirical support for VBN theory at the cognitive level and offers policy-relevant insights for promoting “Bamboo Instead of Plastic” initiatives.

## 1. Introduction

Plastic pollution has emerged as a central challenge in global environmental governance, with its cross-regional diffusion characteristics and long-term ecological hazards drawing significant attention from the international community (UNEP, 2021). In response to this challenge, major economies such as those of the European Union and ASEAN have successively introduced frameworks to ban and limit plastic use (ASEAN, 2021; EU, 2019). In 2020, the Chinese government issued the ‘Opinions on Further Strengthening Plastic Pollution Control,’ which clearly outlined policy directions aimed at reducing plastic consumption and promoting biodegradable alternative materials (China National Development and Reform Commission & China Ministry of Ecology and Environment, 2020). In 2022, it launched the global initiative ‘Bamboo Instead of Plastic’ in collaboration with the International Bamboo and Rattan Organization, and, in 2023, it released the ‘Three-Year Action Plan for Accelerating the Development of ‘Bamboo Instead of Plastic’,’ marking a systematic step by China in promoting the industrialization of plastic alternative materials (China National Development and Reform Commission et al., 2023). Bamboo, as a typical rapidly renewable resource, possesses characteristics such as a short growth cycle (3–5 years), strong carbon sequestration capacity (annual carbon sequestration of 12–15 tons per hectare), excellent mechanical properties, and a low environmental load throughout its life cycle, making it an ideal material choice for plastic alternatives (Lin et al., 2025). Against this backdrop, the consumption of bamboo products, recognized as an important green consumption behavior, ultimately depends on consumers’ awareness and decision-making. However, current research on the market acceptance of bamboo products among consumers and the decision-making mechanisms involved remains insufficient.

Previous research has systematically covered mature product categories, including green agricultural products via studies on the driving factors influencing purchase intentions of green agricultural products in the context of live-streaming e-commerce, and research on the economic impact of consumers’ perceptions of product authenticity on agricultural marketing under green constraints (Sun, 2023; S. Zheng et al., 2023); green home appliances, via studies examining the mechanisms by which energy efficiency labels influence residents’ willingness to purchase energy-saving appliances and research exploring the moderating role of ecological literacy on the purchase intentions for these appliances (Basiru et al., 2024; Rai et al., 2025); and new energy vehicles, via studies investigating consumers’ motivations for purchasing new energy vehicles and research on the impact of various forms of social learning on new energy vehicle sales (Jiang et al., 2025; Thwe et al., 2025). In contrast, bamboo products, as central to the “Bamboo Instead of Plastic” policy, exhibit significant differences in research focus compared with the aforementioned mature categories. Existing research predominantly emphasizes macro-level industrial and technological aspects, such as industrial planning, the relationship between design attributes and consumer satisfaction, and the development of processing technologies and equipment (Lei et al., 2024; Xue et al., 2024; Ye et al., 2024; J. Zhang et al., 2024). However, there is a notable lack of exploration into consumer behavior, particularly concerning the micro-level cognitive mechanisms underlying purchasing decisions. When purchasing green products, consumers must evaluate and consider external attributes such as quality (Z. Liu & Hu, 2022; Luan et al., 2020), price (Ling et al., 2021; Yu et al., 2019), brand (Hu et al., 2024; X. Liu et al., 2025), and packaging materials (Macht et al., 2023; Ollitervo et al., 2025). However, the influence of the core physical attributes of the product itself—specifically, the material—on consumers’ purchase intentions, particularly regarding emerging eco-friendly materials like bamboo, has not been sufficiently addressed. Previous studies have predominantly utilized questionnaires to investigate differences in consumer choice behaviors toward products made from various materials. Given that material perception is reliant on instantaneous sensory integration and value construction, traditional questionnaires are constrained by the retrospective self-report paradigm, which hinders the capture of dynamic cognitive processes. Furthermore, green consumption values have been established as a crucial psychological variable driving green consumption (C. Wang, 2019; J. Wang et al., 2020), making the exploration of their mechanisms of action significantly important. Existing research indicates that green consumption values affect green consumption decisions through various pathways, including an increased emphasis on environmental attributes and the formation of positive product attitudes. Nevertheless, empirical evidence remains lacking on how green consumption values direct consumers’ visual attention processing of the environmental attributes of bamboo materials, thereby influencing purchase decisions.

In light of this, this study focuses on the policy context of “substituting bamboo for plastic” aiming to extend the aforementioned research and make theoretical contributions. Firstly, the existing literature primarily concentrates on mature categories (i.e., green agricultural products, energy-saving appliances, new energy vehicles, etc.) (Basiru et al., 2024; Thwe et al., 2025; S. Zheng et al., 2023), while research on consumer behavior towards bamboo products—an emerging category of green alternatives—particularly regarding the cognitive processing mechanisms of materials, is notably insufficient. This study explicitly centers around the intrinsic material properties of bamboo products, thereby expanding the research on green consumption in the specific context of “substituting bamboo for plastic.” Secondly, this study strives to deepen the application of the value–belief–norm (VBN) theory in specific product decision-making contexts. Although the VBN theory has been proven effective at explaining various environmental behaviors (Carfora et al., 2021; Lee et al., 2023), its operational mechanisms in micro-level decision-making for specific green products (e.g., bamboo products), particularly the “black box” of the cognitive processes linking values to behaviors, still lack empirical exploration. By introducing eye-tracking technology, this study seeks to reveal how green consumption values (GCVs) guide consumers’ selective attention and in-depth processing of products’ environmental attributes, thereby uncovering the micro-mechanism of the “values → cognitive processing → behavior” chain within the VBN theory. The aim is to provide new insights into understanding the consistency between values and behaviors by examining the interaction between product material attributes and green consumption values.

## 2. Literature Review and Hypothesis Development

### 2.1. Green Purchase Intention

Green purchase intention (GPI) reflects consumers’ preferences for environmentally friendly products or services, embodying a combination of their environmental concerns and proactive behavioral tendencies (Srivastava, 2007). The theoretical development of GPI has evolved from a singular focus on environmental concern to a multidimensional integration. Notably, the green product preference model transcends the traditional attitude–behavior research paradigm by conceptualizing consumption decisions as a dynamic matching process between product attributes and individual preferences. It emphasizes that consumers evaluate and weigh the multidimensional attributes of products, including environmental attributes, based on their preferences under the principle of utility maximization (Zhou et al., 2022). Individuals with green preferences incorporate environmental friendliness into their utility functions, thereby achieving a higher perceived value from consuming green products (Bezin, 2019).

Factors influencing consumers’ green purchase intentions can be categorized from the perspectives of both enterprises and consumers, primarily including product factors and individual consumer factors. At the product level, research on core attributes affecting green purchase intentions is relatively mature. The positive effect of perceived quality, as a fundamental attribute, on purchase intention has been widely validated, with its influencing mechanisms explored across various consumption contexts (e.g., residential markets, e-commerce platforms) (Wattoo et al., 2025; Yang et al., 2019). The price attribute has been examined from an interdisciplinary perspective of economics and psychology, forming a multidimensional research framework. For instance, Tan et al. (2025) found that price sensitivity mediates the relationship between perceived quality and green product purchase intention. The study by Z. Zheng et al. (2025) demonstrates that price perception is negatively correlated with consumers’ willingness to purchase energy-efficient air conditioners. Brands influence consumer decision-making through pathways such as green image, communication strategies, and trust-building mechanisms (Hu et al., 2024; Rehman et al., 2025). In contrast, as a core component of a product’s physical attributes, the independent role and influencing mechanisms of material factors in green purchasing decisions still require further exploration.

The influence of consumers’ personal factors on green purchase intention can be categorized into two levels: demographic characteristics and psychological factors. Regarding demographic characteristics, early studies (Levin, 1990) have found that females, younger individuals, higher-income consumers, and those with higher education levels generally exhibit a stronger tendency towards green consumption. However, the explanatory power of demographic variables is significantly limited, as they can account for only 10% to 15% of the variation in green consumption behavior (Witek & Kuźniar, 2020). This limitation has prompted scholars to explore psychological mechanisms in greater depth. Compared with demographic characteristics, psychological factors have been shown to be more robust predictors of green purchase intention. Existing research primarily focuses on general psychological variables that are broadly applicable to the field of environmental behavior, including environmental responsibility and environmental awareness. Environmental responsibility, a crucial dimension of moral identity, enhances green consumption tendencies by activating individuals’ perceived responsibility for environmental damage (Wu & Yang, 2018). Environmental awareness, by shaping individuals’ knowledge structures and risk perceptions regarding environmental issues, positively predicts green consumption attitudes, subjective norms, and perceived behavioral control. These factors collectively mediate the impact on green consumption intention and actual behavior (Xie et al., 2022). Unlike the general psychological variables applicable to various environmental behaviors mentioned earlier, green consumption values (GCVs) form a specific construct proposed by Haws et al. (2014) within the consumption context, focusing on the consumption domain rather than general environmental behavior. GCVs not only pertain to environmental resource conservation but also involve considerations of personal financial and material resources. The authors aimed to “develop a method to understand differences across consumers who do and do not value conserving the environment as part of their consumption behavior,” demonstrating that green consumption values can predict consumers’ preferences for environmentally friendly products. Although scholars have investigated the impact of GCVs on green consumption behavior, the cognitive mechanisms underlying their effects remain to be further explored.

### 2.2. Value–Belief–Norm Theory (VBN Theory)

The value–belief–norm (VBN) theory, proposed by Stren (2000), serves as a comprehensive theoretical framework for explaining pro-environmental behavior. This theory integrates value theory, the new ecological paradigm, and the norm activation model, with the aim of uncovering the psychological driving chain behind individual environmental behaviors. The core logical chain is as follows: an individual’s values shape their beliefs, which, when they perceive environmental threats and their impact on personally cherished matters, activate their personal norms, ultimately driving pro-environmental behavior.

The VBN theory has been extensively utilized to elucidate and forecast various environmental behaviors, showcasing its substantial explanatory power. In the realm of policy support, research conducted by Steg et al. (2005) indicates that the VBN model can effectively anticipate public acceptance of environmental policies. Furthermore, a considerable body of literature has substantiated the explanatory strength of the VBN theory concerning green purchase intentions. For example, Carfora et al. (2021) found that values significantly and positively influence consumers’ intentions to purchase organic food by shaping beliefs and activating personal norms. Lee et al. (2023) demonstrated that the value–belief–norm (VBN) mechanism positively influences consumers’ pro-environmental personal norms, which significantly predict their intention to use electric vehicles. Jebarajakirthy et al. (2024) conducted a meta-analysis based on the VBN framework (including perceived adverse consequences, ascribed responsibility, and personal norms) as well as psychological factors derived from the theory of planned behavior (TPB) (such as subjective norms and attitudes), revealing that altruistic and biospheric values positively and significantly predict psychological factors, ultimately impacting green purchasing behavior. Baek (2024) examined the environmental behavior intentions of meal kit consumers through the lens of VBN theory, finding that values, beliefs, personal norms, and anticipated emotions can enhance these intentions. Pegan et al. (2025) integrated the unified theory of acceptance and use of technology with VBN theory, discovering that Generation Z prioritizes usability and engagement when adopting sustainable consumption assistance applications (SCAAs), whereas Generation Y is more influenced by green consumption values and behavioral norms.

In previous studies, the application of VBN theory has primarily focused on delineating the psychological pathway from values to beliefs, norms, and subsequently to behaviors. Related research has predominantly relied on traditional questionnaire–report paradigms to measure psychological constructs such as beliefs and norms. However, this method struggles to capture real-time, dynamic cognitive activities during the decision-making process, posing risks of retrospective bias and social desirability bias. Moreover, it fails to unveil the real-time processing mechanisms within the ‘black box.’ In light of these limitations, the present study will emphasize the specific micro-cognitive processes (e.g., information attention, perception, processing, and decision evaluation) that connect values with behaviors, elucidating how values guide individuals in allocating cognitive resources (visual attention) within specific decision-making contexts, such as when faced with products made from different materials.

### 2.3. Product Material and Purchase Intention

Material selection stands at the core intersection of product design and sustainable development. Plastics, due to their ease of molding, low cost, and strong plasticity, achieved widespread substitution of traditional materials, such as metals and glass, in the 20th century (e.g., “replacing steel with plastics”), profoundly transforming the manufacturing landscape. However, the non-degradable nature and fossil fuel dependency of plastics have made them significant contributors to global climate change and environmental pollution. In this context, “replacing plastics with bamboo” as an innovative pathway for resource recycling not only provides a material foundation for reducing plastic pollution but also promotes the synergistic sustainable development of resource systems and socio-economy by advancing the substitution of bio-based materials.

The environmental attributes of bamboo constitute its core competitiveness in replacing plastics, specifically manifested in the synergistic effects of its low carbon production and carbon sequestration functions. Compared with plastic and wooden products, bamboo products exhibit lower environmental burdens and higher economic sustainability (Alamerew et al., 2025; Sovbetov, 2025). Life cycle assessment research indicates that although bamboo generates certain carbon emissions during raw material production, processing, and transportation, its carbon storage function offsets the majority of these emissions (Chang et al., 2018; Lao et al., 2025; Vogtländer et al., 2010). M. Zhang et al. (2024) proposed the “Bamboo Instead of Plastic” effect (EBSP) evaluation framework, which demonstrated through three-dimensional indicators—substitution emission reduction rate, material substitution rate, and product renewal ratio—that 30 typical bamboo products could achieve significant environmental benefits by reducing plastic consumption. The energy consumption, particularly fossil energy, in the bamboo manufacturing process is significantly lower than that of plastic. The competitive advantage of bamboo material is not only reflected in its objective environmental benefits but also in its unique sensory experience, which may strengthen the formation mechanism of consumer preferences. B. Wang et al. (2025) indicated that the sensory cues of wood–plastic composites directly influence consumers’ green choices. Chen and Zhang (2015) proposed that consumers’ emotional attachment to natural materials is significantly stronger than to artificial materials. Bamboo, characterized by its fine texture and warm feel, offers a unique visual layering and tactile experience, which starkly contrasts with the industrial aesthetic of plastic.

Multiple empirical studies have further substantiated consumers’ preference for eco-friendly material products and their willingness to pay. The discrete choice experiment conducted by Halton et al. (2022) demonstrated that, in toothbrush purchase decisions, the material of the toothbrush handle had a positive impact on participants’ choices, with bamboo toothbrushes having the highest relative value coefficient. To choose this more environmentally friendly toothbrush, consumers were willing to pay a premium of GBP 4.85 for bamboo products, confirming the advantage of bamboo as a natural and eco-friendly option in consumer choices. Similarly, in the broader field of eco-friendly packaging, Macht et al. (2023) found that consumers’ willingness to purchase bio-based packaging was significantly higher than that of traditional plastic packaging, and this preference was primarily driven by perceived environmental friendliness. Ollitervo et al. (2025) further demonstrated that eco-friendly packaging materials influence purchasing intention through dual pathways of enhancing the product’s perceived attractiveness and environmental friendliness. Although these studies involve different product categories, they all illustrate that the environmental material attributes of products are a critical variable affecting consumer evaluation, preference, and purchasing decisions. Based on the above research, this study proposes the following hypothesis:

**H1.**  *Product material significantly influences consumer purchase intention, with consumers showing a higher purchase intention for bamboo material products compared with plastic material products.*

### 2.4. Green Consumption Values and Purchase Intention

The idea of green consumption values (GCVs), a fundamental construct influencing individual green consumption behaviors, is theoretically grounded at the intersection of environmental psychology and consumer behavior studies. Haws et al. (2014) defined GCVs as “an individual’s inclination to express their environmental values through purchasing and consumption behaviors,” highlighting the orienting and enduring impact of these values on consumption choices. Regarding the mechanisms of green consumption values, existing research has identified multidimensional pathways of influence. Abbey et al. (2015) discovered that consumers with tendencies towards green consumption place greater emphasis on environmental attribute information when evaluating remanufactured products, leading them to perceive such products as more attractive. Furthermore, research has confirmed that GCVs have a significant direct positive effect on pro-environmental consumption intention (J. Wang et al., 2020). Moreover, their influence also operates through indirect pathways, such as by enhancing consumers’ attitudes toward organic clothing, which in turn increases the purchase intention among young consumers (Varshneya et al., 2017). Green consumer values (GCVs) have also been shown to play a significant moderating role. Yan et al. (2021) found that higher levels of GCVs amplify the positive impact of power on green consumption. Ribeiro et al. (2025) demonstrated that, in high-GCV groups, positive attitudes are more likely to translate into actual willingness to pay.

According to VBN theory, GCVs, as specific “self-transcendence” values, constitute the starting point of the chain that drives green consumption decisions. Individuals possessing strong GCVs shape their beliefs regarding environmental issues, which subsequently activate their personal norms, ultimately prompting them to engage in green consumption behaviors, such as purchasing bamboo products. In the consumer decision-making process, green consumption values influence product evaluation through a ‘top-down’ cognitive processing approach, prioritizing environmental attributes in the decision-making framework (C. Wang, 2019). This values-driven mechanism results in individuals with high green consumption values exhibiting a pronounced environmental preference in product selection. They respond to their internal environmental consciousness by selecting green products that align with their environmental motivations. Therefore, the following hypothesis is proposed:

**H2.**  *Green consumption values influence consumers’ purchase intentions, consumers with high green consumption values are more willing to purchase bamboo products compared with those with low green consumption values.*

### 2.5. Eye Movement and Cognitive Processing

Visual attention is a fundamental component of consumer information processing and decision-making. The “Eye-Mind Hypothesis” proposed by Just and Carpenter (1980), suggests that the locations of visual fixation and the foci of cognitive processing are temporally synchronized, indicating that eye movement metrics can directly reflect attention allocation strategies during information processing. Eye-tracking technology, grounded in the “Eye-Mind Hypothesis” provides insights into cognitive processing by recording metrics such as fixation points and saccades. Fixation duration and fixation counts are commonly used as key indicators of visual attention resource allocation. Eye-tracking technology overcomes the limitations of traditional research methods, offering objective and real-time data on cognitive processes in consumer behavior research.

Eye-tracking technology has been applied in multiple areas of consumer behavior research, primarily focusing on the association mechanism between visual attention allocation and purchase decision-making. Consumers are more likely to choose products or brands they have looked at for a longer duration (Lohse, 1997). The study by Janiszewski et al. (2013) found that the more visual attention resources a product receives, the significantly higher the probability of it being chosen. Attention behavior can influence subsequent choices, and there is a certain causal relationship between visual attention and decision outcomes. It may indicate that the stimulus has higher attractiveness, triggering more sustained interest and preference, or it may reflect higher cognitive effort or depth of information processing, such as when the stimulus is novel, complex, or the decision is difficult, requiring individuals to invest more time in understanding and evaluation (Orquin & Loose, 2013; Pieters & Warlop, 1999). Therefore, when interpreting these metrics, especially in the context of relatively novel products, it is necessary to integrate them with other behavioral data, such as purchase intention, for a comprehensive assessment.

Research has confirmed that elements such as packaging design, claim labels, image presentation methods, and layout typography significantly influence purchasing decisions by affecting attention allocation (Bogomolova et al., 2020; Steinhauser et al., 2019; C. Wang, 2023; W. Zhang et al., 2022). In the realm of green consumption, while eye-tracking research is still in its nascent stages, it has already demonstrated unique value. Guyader et al. (2017) found that consumers’ fixation duration on eco-friendly products is positively correlated with their willingness to pay a green premium, and that green price tags assist consumers in locating the eco-friendly products they desire. Najafabadiha et al. (2025) confirmed that environmental education video interventions can enhance the proportion of consumers’ fixation on product environmental information, revealing a consistent preference pattern for eco-friendly products among participants exposed to environmental education information. Based on the value–belief–norm (VBN) theory and the ‘eye-brain linkage’ hypothesis, values, as core psychological constructs, may guide individuals’ selective attention (Just & Carpenter, 1980; Stren, 2000). As a stable values trait, green consumption values may influence consumers’ processing of product environmental attributes by directing selective attention. Individuals with high green consumption values, motivated by intrinsic environmental concerns, may exhibit an automatic attentional bias towards the material characteristics of bamboo products. The material differences between bamboo and plastic products are primarily conveyed through visual perception. Traditional questionnaire surveys often struggle to capture this instantaneous perception–evaluation process; however, eye-tracking technology provides high-resolution data on attention allocation, thereby compensating for the limitations of self-report methods. Understanding the impact mechanism on purchase intention from an eye-tracking perspective holds significant theoretical value. This study aims to achieve two research objectives through eye-tracking technology: (1) to compare the differences in visual attention allocation patterns between bamboo and plastic products among consumers, thereby verifying the influence of product material on visual processing; and (2) to examine how green consumption values moderate these differences in attention allocation, revealing the mechanism of value-oriented cognitive preferences in purchase decision-making. Based on the aforementioned theoretical analysis and literature review, the following research hypotheses are proposed:

**H3.**  *Compared with plastic products, consumers allocate more visual attention resources to bamboo products, manifested as longer fixation durations and more fixation counts.*

**H4.**  *Compared with consumers with low green consumption values, those with high green consumption values allocate more visual attention resources to bamboo products.*

## 3. Research Methodology

### 3.1. Participants

Using G*Power 3.1 to calculate the sample size, and considering the two-way repeated measures ANOVA applicable to this experiment, the projected total sample size required to achieve a statistical power of β = 0.80, with a significance level of α = 0.05 and a medium effect size of Cohen’s f = 0.25, is at least 35 (Faul et al., 2007). Taking into account the possibility of unusable participant data due to failed corrections during the experiment and the need to use the green consumption values scale for grouping, the sample size should be appropriately increased. An online questionnaire was used to invite 150 participants to complete the green consumption values scale (Haws et al., 2014). Based on the scores from the scale, participants were ranked and contacted via phone to invite those scoring in the top 27% (designated as the high green consumption values group) and the bottom 27% (as the low green consumption values group) to participate in an eye-tracking experiment (Kline, 2013). After excluding those with failed corrections and incomplete data, a total of 70 valid participants were obtained, with a male-to-female ratio of 3:4 and an average age of 20.57 ± 1.92 years. All participants had normal recent or corrected vision and no history of colds, headaches, or psychiatric disorders such as schizophrenia. Before the experiment, participants read and signed the “Informed Consent Form” and were provided with corresponding reward upon completion of the experiment.

### 3.2. Experimental Materials

Experimental materials were selected from the “Daily Products” category outlined in China’s Main Product Catalog for Bamboo Instead of Plastic (2023), which was published as part of the Chinese government’s Three-Year Action Plan to Accelerate the Development of “Bamboo Instead of Plastic.” One representative product was chosen from each of the seven sub-categories within daily products as specific subjects for investigation. Actual samples of bamboo and plastic products were collected from multiple online shopping platforms, including Taobao (www.tao.com) and JingDong (www.jd.com), while ensuring the comparability and consistency of the selected products in terms of shape and price to control for variables and guarantee the reliability of the research findings. Using Photoshop 2020, the resolution of all stimulus materials was standardized to 1280 × 1024 pixels, ensuring uniform image size and consistent positioning of the product pictures. The specific experimental materials are listed in Table 1.

### 3.3. Experimental Design

A mixed-design eye-tracking experiment employing a 2 (product material: plastic vs. bamboo) × 2 (green consumption values: high vs. low) factorial structure was conducted. After the experiment, participants were asked to fill out the green consumption values questionnaire again. This questionnaire utilized the six-item, 7-point scale developed by Haws et al. (2014). The total duration of the experimental procedure was approximately 10 min.

Independent variables (IVs):

(a) Product material (within-subjects factor: bamboo vs. plastic)
(b) Green consumption values (between-subjects factor: high vs. low, grouped based on questionnaire scores).

Dependent variables (DVs):

(a) Behavioral level: Purchase intention (measured on a 7-point scale).
(b) Cognitive level: Eye-tracking metrics (fixation duration, fixation count).

### 3.4. Experimental Equipment and Procedure

The experiment was conducted in a soundproofing laboratory. In this study, we used an SMI-RED 500 Hz device (Senso Motoric Instruments, Teltow, Berlin, Germany). Using infrared corneal–pupil reflection technology, this device can record the eye movement index data of participants in real time. The experimental stimulus materials were presented on a 19-inch display with a resolution of 1280 × 1024 pixels. The distance between the screen and the participant’s eyes during the experiment was approximately 60 cm. After explaining the experimental procedure and precautions to the participants, the eye-tracking device was adjusted, and a 9-point calibration was performed. Calibration was considered successful when the visual angle of the eye was calibrated within 1°. The Experiment Center 3.7 and iView X 2.7 software were used for experimental design, stimulus presentation, and data collection, and the Begaze 3.7 software was used for data processing and analysis.

**Figure 1:**
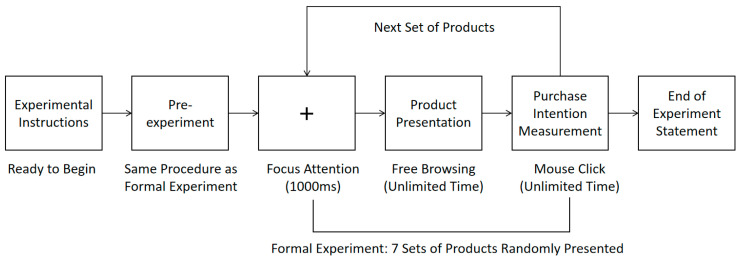
Experimental flow chart.

The experiment is divided into a pre-experiment phase and a formal experiment phase. Initially, the experimental instructions are provided, followed by the pre-experiment, where each participant completes two sets of pre-experimental tasks before proceeding to the seven sets of formal experiments. The materials used in the pre-experiment differ from those in the formal experiment, with the aim of verifying the experimental procedure and ensuring that participants comprehend the task requirements. To better control variables and mitigate the influence of factors such as quality and price, the experimental task is framed as follows: “Next, you will see several sets of different pictures, each containing two products made from different materials. Please disregard their differences in quality and function and make a purchasing decision.” Once the task is clarified, participants press the space bar to access the product interface, where products made from bamboo and plastic are displayed simultaneously. Two products of the same type but constructed from different materials are symmetrically presented on the left and right sides of a single image. The upper section displays the images of the two products, while the lower section provides their respective descriptions, including the product name, material (plastic/bamboo), and price (with both prices being identical). After viewing the product page, participants press the spacebar to proceed to the next page and indicate their purchase intention for the bamboo product on a seven-point scale. If participants understand the experimental procedure, they can commence the formal experiment. During the formal experiment, a total of seven sets of materials are presented, with each experimental material displayed once in a random order. The browsing process is not time-limited; when participants enter the product page, the eye tracker simultaneously records various eye movement metrics, which cease when the spacebar is pressed to advance to the next page. The experiment concludes after all seven sets of products have been presented. The specific experimental procedure is illustrated in Figure 1.

### 3.5. Data Processing

The Begaze software was used for data processing and analysis, and the eye movement indicators and behavioral data of the 70 participants’ product interest areas were exported and analyzed using SPSS 22.0.

## 4. Results

### 4.1. Manipulation Check

Based on the pre-test questionnaire, participants were categorized into two groups: the high green consumption value group and the low green consumption value group, each consisting of 35 participants. An independent samples *t*-test was performed on the green consumption value scores reported by both groups after the experiment. The results indicated that the scores of the high green consumption value group were significantly higher than those of the low green consumption value group (M_high_ = 36.857, M_low_ = 29.171, t = 10.437, *p* < 0.001). This indicates that the manipulation of the green consumption value grouping in this study was effective.

### 4.2. Behavioral Data Analysis

**Figure 2:**
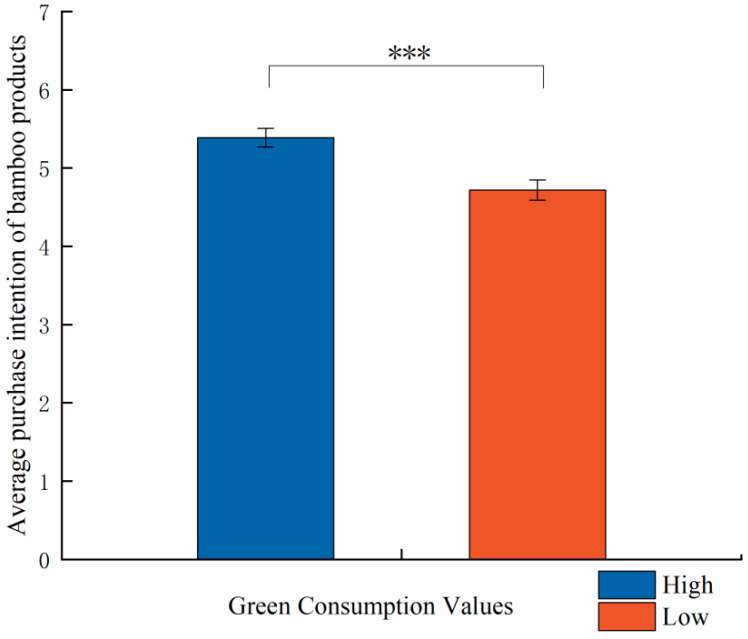
Average purchase intention of bamboo products (*** *p* < 0.001).

The results of participants’ purchase intentions regarding bamboo products are illustrated in Figure 2. It is evident that the average purchase intention for bamboo products exceeds 4 in both the high green consumption values group and the low green consumption values group, indicating a greater inclination among consumers to purchase bamboo products over plastic products. Furthermore, the average purchase intention for bamboo products is significantly higher in the high green consumption values group compared with the low green consumption values group (M_high_ = 5.373, M_low_ = 4.718, t = 3.702, *p* < 0.001). This finding suggests that, when the prices of bamboo and plastic products are equivalent, green consumption values positively influence consumers’ preferences for environmentally friendly options. Thus, hypotheses H1 and H2 are supported.

### 4.3. Analysis of Visual Heatmaps

This study focused on the product image area, maintaining a consistent size throughout the drawing process. The heatmap of the interest area illustrates the position and duration of participants’ visual attention; deeper colors (red) indicate longer fixation times, higher visual hotspots, and greater concentration of attention. Consequently, the heatmaps of the interest areas for the seven product groups were analyzed using BeGaze software. The results, presented in Table 2, indicate that participants exhibited a broader fixation range and longer fixation times for bamboo products compared with plastic products, suggesting that the bamboo products garnered more attention.

### 4.4. Fixation Duration

**Figure 3:**
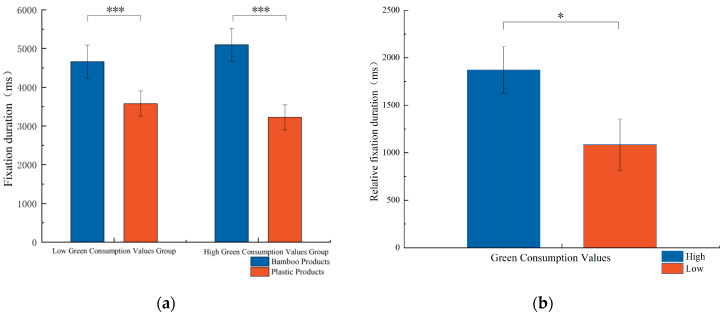
(**a**) Fixation duration and (**b**) relative fixation duration (*** *p* < 0.001, * *p* < 0.05).

With gaze duration as the dependent variable, a two-way repeated measures ANOVA was conducted to analyze the effects of two levels of green consumption values (high vs. low) and two types of product materials (plastic vs. bamboo). The results are presented in Figure 3a. The main effect of green consumption values was not significant. However, the main effect of product material was significant, F(1, 68) = 66.015, *p* < 0.001, η^2^_p_ = 0.493, indicating that participants’ gaze count for bamboo products was significantly higher than that for plastic products. Additionally, the interaction effect between green consumption values and product material was significant, F(1, 68) = 4.670, *p* = 0.034, η^2^_p_ = 0.064. Further simple effects analysis revealed that, under the condition of low green consumption values, participants’ fixation duration on bamboo products (M = 4663.726, 95% CI = [3816.841, 5510.611]) was significantly longer than that on plastic products (M = 3577.903, 95% CI = [2927.336, 4228.470]). Under conditions of high green consumption values, participants’ fixation duration on bamboo products (M = 5098.163, 95% CI = [4251.278, 5945.048]) was significantly longer than that on plastic products (M = 3225.497, 95% CI = [2574.930, 3876.064]). To elucidate the differences in attention allocation among subjects towards products made from different materials and to intuitively observe users’ preferences for these products, the relative fixation duration (i.e., the difference in fixation duration between bamboo and plastic products: T = T_bamboo_ − T_plastic_) was extracted and analyzed. An independent samples *t*-test was performed on the relative fixation duration between the two groups of subjects with high and low green consumption values. The results indicated that the group with high green consumption values had a significantly greater fixation duration compared with the group with low green consumption values (M_high_ = 1872.666, M_low_ = 1085.823, t = 2.161, *p* < 0.05), as illustrated in Figure 3b.

### 4.5. Fixation Counts

**Figure 4:**
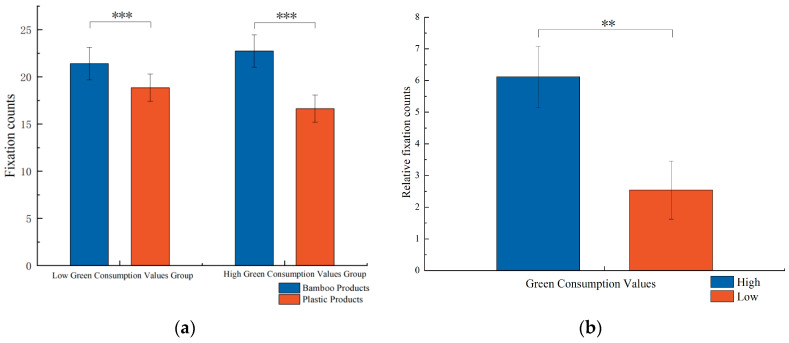
(**a**) Fixation counts and (**b**) relative fixation counts (*** *p* < 0.001, ** *p* < 0.01).

Using the number of fixations as the dependent variable, a two-factor repeated measures ANOVA was conducted with 2 green consumption values (high green consumption values vs. low green consumption values) × 2 product materials (plastic vs. bamboo). The results are illustrated in Figure 4a. The within-subject effect of product material was significant, F(1, 68) = 42.424, *p* < 0.001, η^2^_p_ = 0.384, indicating that the number of fixations on bamboo products was significantly higher than that on plastic products. Additionally, the interaction between green consumption values and product material was significant, F(1, 68) = 7.220, *p* = 0.009, η^2^_p_ = 0.096. Further simple effects analysis was performed. Under low green consumption values, the number of fixations on bamboo products (M = 21.400, 95% CI = [17.962, 24.838]) was significantly higher than that on plastic products (M = 18.857, 95% CI = [15.968, 21.747]). Similarly, under high green consumption values, the number of fixations on bamboo products (M = 22.743, 95% CI = [19.305, 26.181]) was significantly greater than that on plastic products (M = 16.629, 95% CI = [13.739, 19.518]). Thus, hypothesis H3 was supported. The relative fixation count, defined as the difference in the number of fixations on bamboo and plastic products (T = T_bamboo_ − T_plastic_), was analyzed using an independent samples *t*-test for participants in the high and low green consumption values groups. The results indicated that the high green consumption values group had a significantly higher relative fixation count than the low green consumption values group (M_high_ = 6.114, M_low_ = 2.543, t = 2.687, *p* < 0.01), as shown in Figure 4b. Thus, hypothesis H4 was supported.

## 5. Discussion

### 5.1. The Impact of Product Material on Consumers’ Purchase Intention and Visual Attention Processing

The behavioral results of this study clearly indicate that consumers demonstrate a significantly higher purchase intention for bamboo products in comparison to plastic products. This finding strongly corroborates the notion that bamboo products, with their outstanding environmental attributes and unique natural texture, have garnered positive evaluations and preferences in consumer decision-making. As the core carrier of the “Bamboo Instead of Plastic” policy, bamboo material, characterized by its rapid renewability, low carbon footprint, and biodegradability, significantly enhances consumers’ subjective judgments regarding the product’s positive environmental contributions. The perception of a product’s environmental benefits serves as a key antecedent variable driving consumers’ preferences for and purchase intentions towards eco-friendly products (Wan et al., 2024). The bamboo material itself conveys a strong signal of environmental friendliness, effectively enhancing consumers’ perception of environmental benefits, which in turn translates into stronger purchase intentions. Yao’s (2023) study revealed the impact of product display methods on green consumption, finding that showcasing products based on green attributes or utilizing green category labels can effectively guide consumer choices. In this study, bamboo material itself serves as an intuitive and intrinsic “green category label,” enhancing consumers’ recognition of the product’s environmental attributes and triggering positive green associations and evaluations without relying on additional labels, thereby promoting the formation of purchase intentions.

Further eye-tracking data provided critical evidence for understanding the micro-cognitive mechanisms underlying this decision-making process. The results revealed that, compared with plastic products, consumers exhibited longer fixation durations and higher fixation counts when evaluating bamboo products. This indicates that consumers allocated more visual attentional resources and engaged in deeper cognitive processing while handling information about bamboo products. Longer fixation durations typically suggest that the stimulus possesses greater attractiveness or elicits more sustained interest (Janiszewski et al., 2013). In the context of this study, bamboo products—due to their unique environmental values, natural and warm sensory experience, and potential relative scarcity—were more likely to capture consumers’ attention and encourage them to conduct active and detailed evaluations. This finding aligns with the research by H. Wang et al. (2023) on food packaging materials, which noted that eco-friendly materials attract more cognitive resources and are associated with positive consumption tendencies. The significantly higher purchase intention results strongly support this interpretation: consumers are willing to spend more time scrutinizing bamboo products, ultimately demonstrating a stronger purchase inclination. This suggests that such attentional investment is likely driven by interest and positive evaluation motivations.

On the other hand, the increase in fixation duration and frequency may also indicate heightened cognitive effort, particularly when the stimuli are novel, complex, or when decision-making involves uncertainty (Pieters & Warlop, 1999). For relatively emerging bamboo material products—especially in complex categories such as bamboo-shell electronic products and bamboo-based composite utensils—consumers may require additional information processing and comprehension. This ‘need for comprehension’ may partially explain the prolonged fixation duration. However, considering the strong purchase intention observed at the behavioral level, the cognitive effort associated with ‘novelty’ or ‘uncertainty’ may not be the primary factor influencing this study. If the increase in fixation duration and frequency primarily arises from cognitive difficulty or uncertainty that is challenging to comprehend, it would typically not be accompanied by, and may even inhibit, higher purchase intention.

### 5.2. The Impact of Green Consumption Values on Consumers’ Purchase Intention and Visual Attention Processing

The behavioral data from this study indicate that participants in the high green consumption values (GCVs) group demonstrate a significantly greater intention to purchase bamboo products compared with those in the low GCVs group. This finding is consistent with existing research that suggests GCVs form a driving factor in green consumption behavior (J. Wang et al., 2020). Based on the value–belief–norm (VBN) theoretical framework, GCVs constitute the starting point of the chain that drives green consumption decision-making. Individuals with a stronger GCVs are influenced by their intrinsic environmental value orientation, which leads them to develop beliefs about environmental issues, such as acknowledging the environmental benefits of bamboo material. These beliefs subsequently activate personal norms that encourage environmentally responsible actions, including the selection of eco-friendly products. Ultimately, this activated personal norm results in a heightened intention to purchase bamboo products (Baek, 2024; Carfora et al., 2021; Stren, 2000). This study offers new empirical evidence supporting the application of VBN theory in elucidating the decision-making processes involved in the purchase of specific environmentally friendly products, such as bamboo.

Eye-tracking data further elucidates the micro-cognitive mechanisms through which green consumption values (GCVs) influence decision-making. The study found that participants in the high GCVs group allocated significantly longer relative fixation durations and higher relative fixation counts to bamboo products. This allocation of visual attention resources reflects intrinsic cognitive processing. Research conducted by Sheng et al. (2019) indicates that consumers with positive environmental attitudes prioritize the high environmental value of green products and consider the potential adverse effects of their purchasing behavior on the environment when making buying decisions. Individuals with elevated green consumption values are more readily attracted to the environmental value of products, which they regard as a key factor in product evaluation and selection. Additionally, research by C. Wang et al. (2020) on visual processing indicates that longer fixation durations and a greater number of fixations on the area of interest correlate with increased interest in the content or product within that area. Green consumption values, as a core psychological structure, guide individuals’ selective attention. Consumers with high GCVs exhibit an automatic attentional bias toward the environmental attributes of products. Bamboo material, as a salient environmental cue, can more effectively capture and sustain visual attention among individuals with high GCVs, thus becoming a focal area for information processing.

Although the main effect of green consumption values (GCVs) did not achieve statistical significance in the overall eye-tracking analysis, its interaction with product material was significant. When presented with bamboo products, individuals with high GCVs allocated a disproportionately greater amount of visual attention resources, as evidenced by longer relative fixation durations and higher fixation counts, compared with those with low GCVs. This finding suggests that the influence of GCVs is not independent; rather, it manifests through its interaction with the product’s environmental attributes, specifically material. The values held by high-GCV consumers enhance their visual attention bias towards products with salient environmental attributes. The product material (bamboo versus plastic) serves as the primary factor driving the overall differences in visual attention, while GCVs modulate the extent of this difference, particularly by increasing attentional investment in bamboo products.

## 6. Conclusions

This study, grounded in the value–belief–norm Theory, employs eye-tracking technology to explore consumers’ decision-making processes regarding the purchase of bamboo-based products in the context of substituting bamboo for plastic. The research focuses particularly on the influence and interaction between product material and green consumption values (GCVs). At the behavioral level, the findings indicate that consumers exhibit a significantly higher intention to purchase bamboo-based products compared with plastic alternatives. This suggests that the environmental attributes and natural texture of bamboo products are widely recognized and preferred by consumers. Furthermore, consumers with high GCVs demonstrate a markedly stronger intention to purchase bamboo-based products than those with low GCVs, confirming that GCVs effectively motivates consumers to select products that align with their values.

At the cognitive processing level, eye-tracking results reveal that, when compared with plastic products, consumers allocate more visual attention resources when evaluating bamboo products, specifically manifested as longer fixation durations and higher fixation counts. Moreover, individuals with strong green consumption values pay more attention to bamboo products, something which is coupled with significantly higher purchase intentions. This increase in attentional resources reflects consumers’ interest, emphasis, and positive evaluation of bamboo products, attributed to their environmental attributes, unique texture, and potential scarcity. Although the need to understand the relatively novel characteristics of bamboo material may contribute to some cognitive effort, behavioral results support the notion of “interest-driven positive evaluation” as the dominant explanation.

## 7. Theoretical Contributions and Practical Implications

### 7.1. Theoretical Contributions

Current research on green consumption predominantly focuses on well-established product categories, such as green agricultural products, energy-saving appliances, and new energy vehicles. However, there has been insufficient attention given to bamboo products, which are pivotal to the “Bamboo Instead of Plastic” policy. This study specifically targets this emerging and strategically significant category of green substitutes, systematically examining consumers’ cognitive responses and decision-making preferences regarding its core physical attribute—material. This not only addresses the urgent need for global plastic pollution control but also enriches the research on green consumption, particularly in the context of ‘replacing plastic with bamboo.’ It provides critical empirical evidence for understanding the market acceptance mechanisms of emerging green products.

This study integrates green consumption values (GCVs) with eye-tracking technology to explore the intricate processes through which values influence consumption decisions. It enhances the application of the value–belief–norm (VBN) theory in specific product decision-making contexts, providing robust support for the VBN theory’s applicability in explaining the purchase decisions related to specific green products, such as bamboo products. This research offers direct evidence for the VBN theory chain in micro-level, specific product selection scenarios. A significant theoretical contribution of this study is its revelation of the micro-pathway through which GCVs influence purchase decisions by guiding the allocation of visual attention. Eye-tracking data reveal that high-GCV consumers exhibit a significant visual attention bias towards bamboo products, characterized by longer fixation durations and increased fixation counts. This “top-down” selective attention mechanism allows bamboo material, as a key environmental cue, to be preferentially captured and sustained within cognitive resources, thereby promoting positive product evaluations and stronger purchase intentions. This research bridges the gap between values studies and micro cognitive neuroscience, offering a novel and refined perspective on how values specifically guide consumer behavior. The study identified a significant interaction between GCVs and product material (bamboo vs. plastic). High GCVs notably amplify consumers’ visual attention investment and purchase preference for bamboo products, indicating that the environmental attributes (material) of the product itself are key contextual factors in activating and strengthening “value-behavior consistency.”

This research introduces the eye-tracking experimental paradigm into the field of bamboo product consumption research, thereby overcoming the limitations of traditional questionnaire methods in capturing dynamic and implicit cognitive processes. By quantitatively analyzing consumers’ subconscious visual preferences for products made from different materials, this research empirically verifies the significant effect of material attributes on consumer product evaluation. It provides objective physiological evidence supporting the assertion that “product physical attributes (material) directly influence decision-making,” thereby enriching the theory of green product design. Furthermore, it advances green consumption research from the macro behavioral level to the micro cognitive level, laying a methodological foundation for the subsequent exploration of consumers’ information processing patterns regarding green attributes, such as information search strategies and decision conflicts.

### 7.2. Practical Implications

In this paper, utilizing eye-tracking technology, has revealed how green consumption values (GCVs) influence consumers’ purchase intentions by guiding their visual attention allocation in the context of “Replacing Plastics with Bamboo.” The discovery of this micro-cognitive mechanism provides practical guidance for the Chinese government in promoting the “Replacing Plastics with Bamboo” policy, as well as for enterprises marketing bamboo-based products. The research confirms that GCVs are a crucial psychological variable driving both the purchase intentions for bamboo products and visual attention. The government can implement a tiered communication strategy. For the high-GCV group, the content can focus more on the deeper environmental values of bamboo materials (such as carbon sequestration capacity and low carbon footprint throughout their lifecycle), reinforcing their intrinsic value recognition. For the low-GCV group or the general public, the emphasis can be on popularizing the immediate hazards of plastic pollution, the renewable and biodegradable characteristics of bamboo materials, and the instant environmental benefits of “replacing plastic with bamboo” (such as the reduction of white pollution), gradually guiding attention and behavioral shifts by enhancing environmental awareness.

Eye-tracking results indicate that bamboo material effectively captures consumers’ visual attention, suggesting that the government should encourage and support businesses to clearly and prominently showcase the natural characteristics of bamboo (such as texture and feel), making it a natural “green category label.” In product design and packaging, maximize the display of bamboo’s natural beauty and uniqueness (e.g., high-definition images, window designs showcasing texture), avoiding excessive processing that obscures its natural features. Marketing content should emphasize emotional resonance and value alignment, exemplified by phrases like “Choose bamboo, choose a sustainable future,” which narrate bamboo’s environmental story and cultural connotations such as resilience and nobility. Additionally, encouraging users to share their experiences can leverage word-of-mouth to influence low-GCVs audiences. To address potential consumer concerns, it is vital to provide clear and accessible product knowledge, usage and maintenance guidelines, and interpretations of safety labels. This approach actively reduces cognitive barriers and uncertainties, transforming potential “cognitive effort” into positive purchase intentions. Furthermore, promoting the establishment of a unified bamboo sustainability certification label and ensuring its prominent display on products can facilitate quick consumer recognition and emphasize environmental attributes.

Although bamboo products possess unique advantages due to their eco-friendly characteristics and natural texture, they still face several challenges. A certain level of environmental impact persists throughout their entire life cycle, including planting, production, processing, and transportation. Therefore, it is necessary to further enhance the environmental benefits of bamboo products from a life cycle perspective. For bamboo products that come into contact with food, special attention should be paid to quality supervision and compliance with safety standards, emphasizing the need to strengthen the formulation and oversight of safety standards for such products. Additionally, some bamboo-based composite products may lead to consumer misunderstandings regarding their actual composition, making it essential to clearly label the specific ingredients and their proportions in the product description. Recyclability is also a critical issue, especially for electronic products with bamboo shells and bamboo-based composite products. It is crucial to prioritize the establishment of a classification, recycling, and circular utilization system to address concerns about end-of-life treatment, enhance the environmental image of products throughout their lifecycle, and achieve differentiated processing for various “bamboo instead of plastic” products.

## 8. Limitation and Future Research

### 8.1. Limitation

This paper has several limitations that warrant consideration in future research. The participants were predominantly young Chinese consumers, whose values regarding green consumption, cultural backgrounds, and perceptions of bamboo materials may be uniquely specific. Consequently, this somewhat restricts the generalizability of the research findings to broader age groups, diverse regions, and cross-cultural contexts. Additionally, the experiment utilized static product images as stimuli, which failed to adequately replicate the dynamic factors present in real shopping scenarios, such as product quality, functionality, tactile perception, price fluctuations, and interactions with brand information. This limitation may have implications for the ecological validity and external applicability of the research results. Furthermore, the study employed an eye-tracking experiment to examine the role of product material and green consumption values. While some findings related to visual cognition were identified, the investigation did not deeply explore the neural mechanisms underlying consumer behavior during the purchasing process.

### 8.2. Future Research

Future research could expand this topic from multiple dimensions. Exploring more refined eye-tracking metrics—such as changes in pupil diameter, first fixation duration on specific areas of interest, and saccade path patterns—alongside the integration of neuro technologies like electroencephalography (EEG), could enhance our understanding of the micro-cognitive and neural mechanisms underlying green consumption decisions. Additionally, collecting samples from diverse age groups and regions through questionnaire surveys would improve the generalizability of the findings. In the research design, systematically manipulating or measuring product “novelty” and consumer “familiarity” levels would allow one to empirically assess the influence of cognitive effort and comprehension demand factors on visual attention allocation and final decision-making at various stages of product cognition. Furthermore, incorporating more product attributes and psychological variables would allow for a systematic analysis of the factors and mechanisms affecting consumers’ purchase intentions regarding bamboo products. Attention should also be directed towards the interaction between policies and the market, examining how subsidy policies influence the price competitiveness of bamboo products, as well as how supply chain transparency impacts consumers’ trust and purchase intentions. Lastly, designing a virtual market environment could simulate the effects of varying policy intensities (e.g., carbon tax, subsidies) on consumers’ visual search strategies. These research directions will provide more precise guidance for policy formulation and corporate practices.

## Figures and Tables

**Table 1:** Experimental material samples.

Category	Experimental Material Samples						
Product Number	1	2	3	4	5	6	7
Product Category	Bamboo Shell	Bamboo Stationery	Bamboo Personal Care Products	Bamboo Furniture	Disposable Bamboo Tableware	Durable Bamboo Catering Utensils	Bamboo Degradable Bags
Bamboo Products	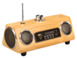	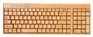	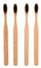	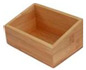	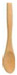	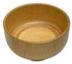	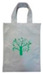
Plastic Products	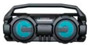	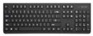	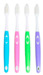	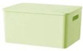	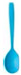	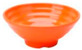	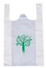
Price (CNY)	100	50	10	10	0.5	5	1

**Table 2:** Heatmap of the area of interest.

Material	Material 1	Material 2	Material 3	Material 4	Material 5	Material 6	Material 7
Bamboo	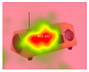	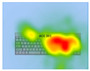	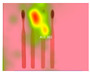	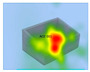	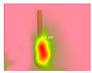	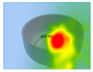	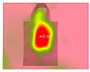
Plastic	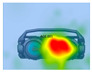	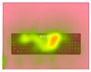	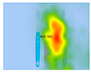	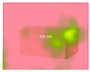	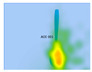	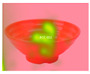	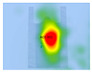

## Data Availability

The data are available from the authors upon reasonable request.
